# MLFGCN: short-term residential load forecasting via graph attention temporal convolution network

**DOI:** 10.3389/fnbot.2024.1461403

**Published:** 2024-09-23

**Authors:** Ding Feng, Dengao Li, Yu Zhou, Wei Wang

**Affiliations:** ^1^College of Computer Science and Technology (College of Data Science), Taiyuan University of Technology, Taiyuan, China; ^2^College of Computer Science and Technology, Taiyuan Normal University, Jinzhong, China; ^3^Shanxi Energy Internet Research Institute, Taiyuan, China; ^4^Key Laboratory of Big Data Fusion Analysis and Application of Shanxi Province, Taiyuan, China

**Keywords:** load forecasting, multi-level feature fusion, neural network, time-series forecasting, graph neural networks

## Abstract

**Introduction:**

Residential load forecasting is a challenging task due to the random fluctuations caused by complex correlations and individual differences. The existing short-term load forecasting models usually introduce external influencing factors such as climate and date. However, these additional information not only bring computational burden to the model, but also have uncertainty. To address these issues, we propose a novel multi-level feature fusion model based on graph attention temporal convolutional network (MLFGCN) for short-term residential load forecasting.

**Methods:**

The proposed MLFGCN model fully considers the potential long-term dependencies in a single load series and the correlations between multiple load series, and does not require any additional information to be added. Temporal convolutional network (TCN) with gating mechanism is introduced to learn potential long-term dependencies in the original load series. In addition, we design two graph attentive convolutional modules to capture potential multi-level dependencies in load data. Finally, the outputs of each module are fused through an information fusion layer to obtain the highly accurate forecasting results.

**Results:**

We conduct validation experiments on two real-world datasets. The results show that the proposed MLFGCN model achieves 0.25, 7.58% and 0.50 for MAE, MAPE and RMSE, respectively. These values are significantly better than those of baseline models.

**Discussion:**

The MLFGCN algorithm proposed in this paper can significantly improve the accuracy of short-term residential load forecasting. This is achieved through high-quality feature reconstruction, comprehensive information graph construction and spatiotemporal features capture.

## 1 Introduction

With the development of society, human demand for electricity is constantly increasing, among which residential electricity consumption is increasing rapidly. According to the World Energy Outlook 2023 (IEA, 2023), residential electricity consumption accounts for 23% of the world’s total annual electricity consumption, with a growth rate faster than any other energy consumption, and is expected to exceed 45% by 2050. The growing residential load is becoming increasingly important to maintain a balance between electricity supply and demand (Afzalan and Jazizadeh, 2019). In the electricity market, residential load forecasting is crucial for decision-makers to carry out activities such as electricity planning, pricing, power quality assessment and customer behavior analysis (Heydari et al., 2020; Rafati et al., 2020).

**Figure 1:**
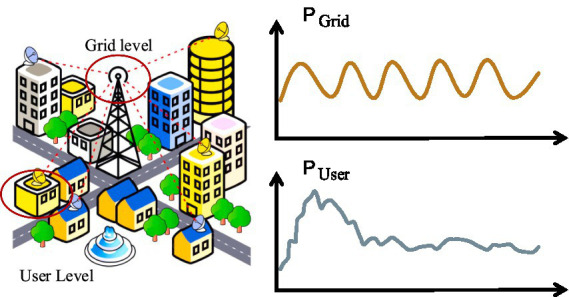
The difference between grid-level and user-level load.

With the introduction of the concept of energy Internet, smart solutions such as smart cities, smart grids are constantly promoted. The deployment of new energy equipment, various flexible loads and new energy vehicle charging piles has made it increasingly difficult to maintain a balance between supply and demand in the power grid. Accurate residential load forecasting is the effective way to solve this problem. Residential load forecasting is to explore changing patterns of residential electricity demand and forecast the load values of a certain period in the future, which is crucial for stable operation of the power system. It has been researched for decades and involves various aspects. Among them, short-term residential load forecasting is the key to analyzing user-side demand and provides an important guarantee for the development of daily power generation planning and the safe operation of the power grid. However, compared with grid-level forecasting, residential electricity consumption has higher uncertainty. As shown in Figure 1, the curve of grid-level load is relatively gentle and has strong regularity, which leads to the forecasting more easier. However, for the user-level electricity consumption, the load curve of a single house has strong volatility due to the differences in user lifestyle habits. The uncertainty and randomness make accurate short-term residential load forecasting more challenging (Yang et al., 2022; Yamasaki et al., 2024; Tan et al., 2023), which is the focus of this study.

Short-term residential load forecasting has been studied for decades as a category of time series forecasting. However, existing time series forecasting methods only use temporal features for prediction and cannot fully explore the valuable information in the data. Recent researches have found that there is a certain correlation between different residential load series, which can be utilized to improve the accuracy of load forecasting. With the development of graph neural networks (GNNs), spatiotemporal load forecasting methods based on GNNs have attracted much attention. Although the prediction accuracy has been significantly improved by introducing GNN based models, there are still some shortcomings. Firstly, existing prediction algorithms mainly focus on improving the model structure to more effectively extract the spatiotemporal features of load data, while ignoring the construction of input features. In the process of load forecasting, the construction of input features enable models to capture potential multi-level dependencies in load data more effectively. Secondly, there is a lack of effective graph construction that includes comprehensive and multi-perspective information when learning spatial features.

To address the aforementioned issues, we propose a novel multi-level feature fusion model based on graph attention temporal convolutional network (MLFGCN) for short-term residential load forecasting. The proposed MLFGCN reconstructs the input load data to better capture the periodic, temporal and spatial dependencies. Additionally, we design two types of adjacency matrices to construct a multi-level information graph, which enables the forecasting model to capture features of load data more comprehensively. The main contributions of this paper are as follows:

- We design an feature reconstruction mechanism for input load series considering the temporal correlations and periodic characteristics of the load data. High-quality feature matrix is obtained by feature reconstruction mechanism, which effectively improves the learning ability of the model. For the input data, two types of adjacency matrices are designed to learn the potential multi-level dependencies in load series. Compared with the traditional Euclidean-based adjacency matrix, we introduce fast dynamic time warping (fastDTW) algorithm to generate the similarity adjacency matrices of individual houses and multiple houses, respectively.
- A novel multi-level feature fusion model based on graph attention temporal convolutional network is proposed for short-term residential load forecasting. TCN with gating mechanism is introduced to learn potential long-term dependencies in the original load data. Two graph attention convolutional modules are then designed to capture potential multi-level dependencies. Finally, the outputs of each module are fused through an information fusion layer to obtain the highly accurate forecasting results.
- We conduct validation experiments on two real-world datasets, which demonstrate that our proposed model is always better than the baselines.

The remainder of the paper is organized as follows. Section 2 provides a discussion of related work. In Section 3, we present the framework of our proposed method in detail. The experimental setup and analysis are described in Section 4. Finally, Section 5 provides the conclusion.

## 2 Related work

**Figure 2:**
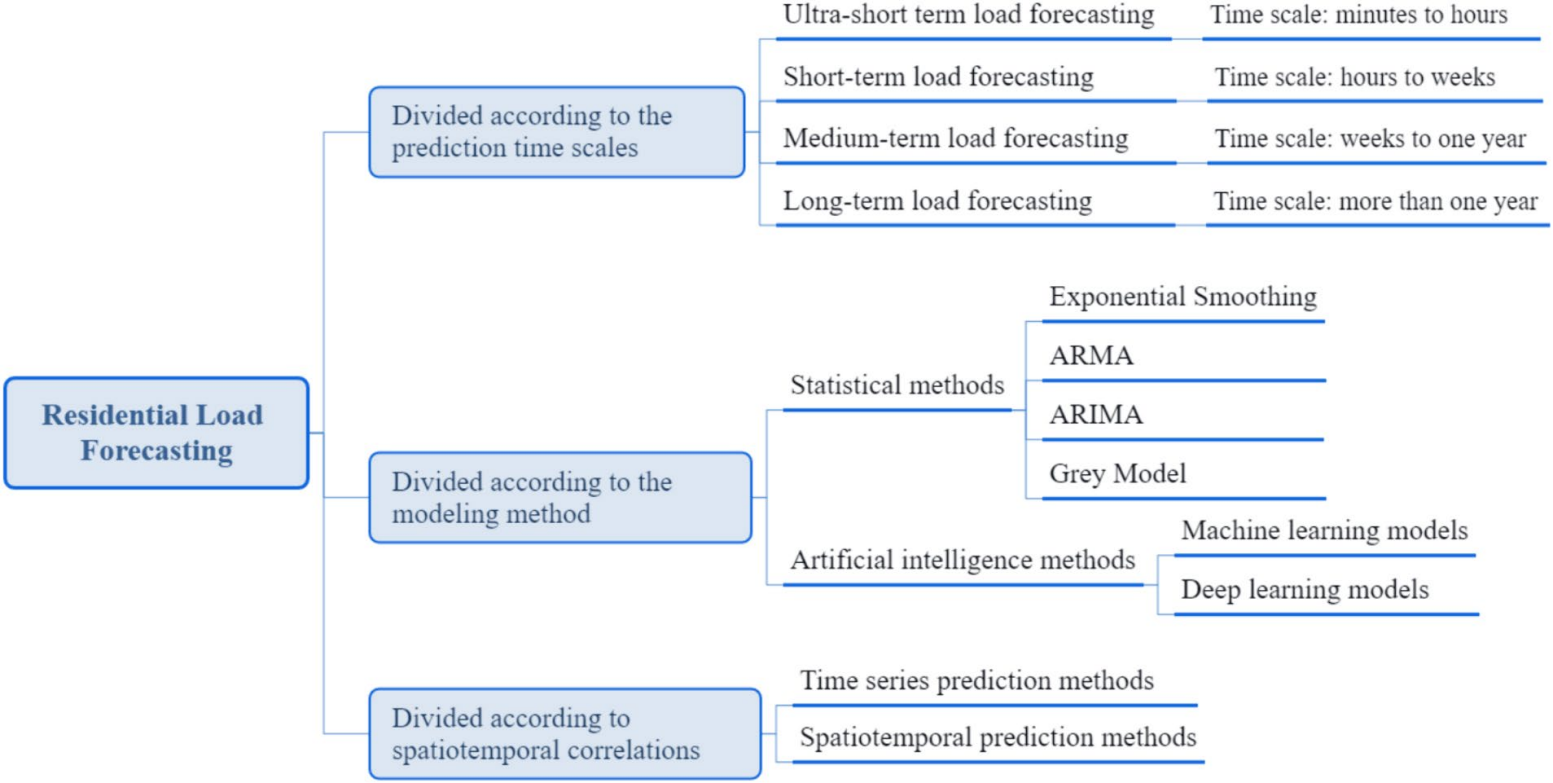
Classification of residential load forecasting methods.

Residential load forecasting is more challenging due to its high randomness and volatility, which is very different from grid-level load forecasting (Zheng et al., 2019). In recent years, many residential load forecasting methods have been proposed, which can be divided from different perspectives (as shown in Figure 2). From the perspective of forecasting time scale, residential load forecasting can be divided into ultra short-term forecasting, short-term forecasting, medium-term forecasting and long-term forecasting. From the perspective of modeling method, residential load forecasting can be divided into statistical models and artificial intelligence (AI) forecasting models. From the perspective of spatiotemporal correlations, residential load forecasting methods can be divided into time series forecasting based methods and spatiotemporal forecasting based methods. In this study, we mainly focus on short-term residential load forecasting based on spatiotemporal forecasting methods.

### 2.1 Time series forecasting

Short-term residential load forecasting has been studied as a time series forecasting problem for many years. The traditional forecasting methods include statistical methods, such as exponential smoothing, auto-regressive moving average (ARMA) (Moon et al., 2021), auto-regressive integrated moving average (ARIMA) (Mahia et al., 2019), and gray model, etc. This type of models is relatively simple, but its accuracy for nonlinear prediction tasks is limited. In recent years, machine learning methods have shown their superiority in capturing temporal correlations and strong generalization ability. Introducing machine learning into load forecasting field can greatly improve the forecasting accuracy (Singh and Mohapatra, 2021; Xia et al., 2023). Shi et al. (2017) proposed a novel pooling-based deep recurrent neural network (DNN) for household load forecasting, which can address the over-fitting problem by increasing the diversity and volume of data. This work made the first attempt to explore the feasibility of deep learning in the application of individual load forecasting and achieved good prediction results. The experimental results showed that the proposed method outperforms SVR by 13.1%, ARIMA by 19.5% and classical deep RNN by 6.5% in terms of RMSE. Chen et al. (2022) proposed a new multi-cycle self-augmented neural network (MultiCycleNet) for household short-term load forecasting. MultiCycleNet learns user’s electricity consumption mode by considering the circular correlation in the load profiles to obtain more accurate forecasting results. The work is the first to use relevant load series considering contextual information from historical data for feature learning of household electricity consumption pattern. The experiments on two publicly available datasets show that the proposed framework outperforms the baselines by 11.14, 9.02, 19.83 and 10.46% in terms of, MAE, MAPE, MSE, and RMSE, respectively. In the recent studies, Transformer-based time series forecasting models have also been introduced into short-term load forecasting research. Ran et al. (2023) proposed a hybrid model incorporating decomposition techniques and Transformer for short-term load forecasting. The proposed model used the mode decomposition techniques to decompose the load data into multiple subseries. Then, these subseries are calculated by sample entropy and recombined based on the principle of combining similar values. The recombined subseries are input into the Transformer model to obtain the final prediction results. Although methods based on time series forecasting have greatly improved the accuracy of short-term residential load forecasting, they mainly focused on temporal correlation (e.g., historical load and weather information) and do not fully consider the spatial correlation of load series.

### 2.2 Spatiotemporal load forecasting

Recently, some researchers found that the load distribution of different houses also have a high spatial correlation, so the concept of spatial dependence was introduced into short-term load forecasting (Yin and Xie, 2021; Liu and Chen, 2021; Jalali et al., 2021). Tascikaraoglu and Sanandaji (2016) the potential spatial correlation between the electricity load of target house and surrounding houses has been mined and used to improve the accuracy of load forecasting. Sajjad et al. (2020) proposed a hybrid residential load forecasting model combining convolution neural network (CNN) and gated recurrent units (GRU). In the proposed CNN-GRU model, CNN are introduced to extract the spatial features of the input load data. The output of CNN are fed into GRU to get the final forecasting results. Although CNN is an effective model for extracting spatial features, it cannot handle non-Euclidean structure data. It is obvious that users with similar geographical locations may have similar electricity consumption patterns due to the similar external environments and holiday effects. Furthermore, users who are geographically far apart but have similar living habit may also have similar electricity consumption patterns. Therefore, methods based on non-Euclidean distance are more suitable for learning the spatial dependencies in load sequences. Recently, the GNN has attracted much attention due to its powerful capabilities in modeling and feature extraction of non-Euclidean structured data. The spatiotemporal forecasting models based on GNN have been successfully applied in load forecasting (Wang et al., 2022; Wang et al., 2022; Feng et al., 2022). Lin et al. (2021) proposed a spatial–temporal short-term load forecasting model based on GCN. The proposed model adopted self-adaptive graph waveNet framework, which was originally designed for audio generation (Oord et al., 2016). For the proposed model, spatial correlations in load series are captured by GCN with self-adaptive adjacency matrix, temporal correlations are learned by TCN. This work is the first attempt to introduce GCN to capture spatial–temporal correlations in electric load. Cheung et al. (2021) the spatial–temporal GCN (STGCN) method was adopted to capture the spatial and temporal correlations in load data for more accurate forecasting results. Experimental results on dataset collected in Iowa showed that the proposed model exhibited significantly better performance in real load prediction than other baselines. Wei et al. (2023) proposed a novel spatial–temporal embedding GNN (STEGNN) for short-term load forecasting. The proposed model first constructed the directed static graphs and directed dynamic graphs. Then, exponential moving average and GCN are combined to capture the spatial and temporal correlations to obtain accurate load forecasting results. Table 1 summarizes and compares the relevant forecasting methods.

**Table 1:** Methods comparison between this study and related works.

Literature	Methodology	Application scenarios	Main contributions
Time series forecasting based methods			
Forecasting Electricity Consumption using ARIMA Model (Mahia et al., 2019)	ARIMA models with different sets of parameters	Forecasting of electricity consumption	This paper proposed ARIMA models with different sets of parameters for forecasting electricity consumption. Best model with estimated parameters, is selected based on the prediction performance.
Deep learning for household load forecasting-A novel pooling deep RNN (Shi et al., 2017)	PDRNN	Household short-term load forecasting	This work made the first attempt to explore the feasibility of a cutting edge algorithm, deep learning, in the application of individual load forecasting and solved the over-fitting problem by increasing the diversity and volume of data.
MultiCycleNet: multiple cycles self-boosted neural network for short-term electric household load forecasting (Chen et al., 2022)	MultiCycleNet	Household short-term load forecasting	The work is the first time to use correlative load series considering contextual information from historical data for feature learning of household power consumption pattern.
Short-term load forecasting based on CEEMDAN and Transformer (Ran et al., 2023)	CEEMDAN-SE-TR	Short-term load forecasting	This paper proposed a hybrid model incorporating decomposition techniques and Transformer for short-term load forecasting. The proposed model first construct the directed static graph and directed dynamic graphs. Then, exponential moving average and GCN are combined to capture the spatial and temporal correlations to obtain accurate load forecasting results.
Spatiotemporal forecasting based methods			
A novel CNN-GRU-based hybrid approach for short-term residential load forecasting (Sajjad et al., 2020)	CNN-GRU	Residential short-term load forecasting	This paper proposed a hybrid residential load forecasting model combining CNN and GRU. In the proposed CNN-GRU model, CNN are introduced to extract the spatial features of the input load data. The output of CNN are fed into GRU to get the final forecasting results.
Spatial–temporal residential short-term load forecasting via graph neural networks (Lin et al., 2021)	Ada-GWN	Residential short-term load forecasting	This work made the first attempt to introduce GNN to capture spatial–temporal correlations in electric load forecasting and proposed a spatial–temporal short-term load forecasting model based on Graph WaveNet framework.
Leveraging Spatial Information in Smart Grids using STGCN for Short-Term Load Forecasting (Cheung et al., 2021)	STGCN Multi-hop	Short-term load forecasting for customers	This work introduced spatial–temporal GCN (STGCN) method to capture the spatial and temporal correlations in load data for more accurate forecasting results
Short-term load forecasting using spatial–temporal embedding graph neural network (Wei et al., 2023)	STEGNN	Short-term load forecasting	This work proposed a novel spatial–temporal embedding GNN (STEGNN) for short-term load forecasting.
Our work	MLFGCN	Residential short-term load forecasting	We propose a novel multi-level feature fusion model based on graph attention temporal convolutional network (MLFGCN) for short-term residential load forecasting. The proposed MLFGCN reconstructs the input load data to better capture the periodic, temporal and spatial dependencies. Additionally, we design two types of adjacency matrices to construct a multi-level information graph, which enables the forecasting model to capture the features of load data more comprehensively.

## 3 Methodology

Based on the analysis of residential load data, this study proposes MLFGCN model for short-term residential load forecasting. MLFGCN learns potentially dependence from historical load data to obtain high-accuracy future load values without any additional information.

### 3.1 Problem formulation

We can represent the residential network as a graph $G=\left(V,E,A\right)$ , where $V=\{v_{1},v_{2},\cdots ,v_{N})$ is the set of all houses, *N* is the number of the houses and *E* is the set of edges. The correlation between houses is represented by adjacency matrix *A*, and $A\in {\mathbb{R}}^{N\times N}$ . In this paper, we use two types of adjacency matrices to learn multi-level interdependence, that is, the self-similarity adjacency matrix $A^{s}$ and the cross-similarity adjacency matrix $A^{c}$ . $A^{s}$ is obtained by calculating the internal similarity of a single load series and $A^{c}$ is obtained by calculating the similarity between any two load series.

We take the historical load data $X=\left[X^{1},X^{2},\cdots X^{N}\right]\in {\mathbb{R}}^{N\times T}$ as the input data, where *T* is the input length of the historical load data. Thus, the aim of short-term residential load forecasting is learning a mapping function f from previous *T* steps load data to the next step load values. It can be defined as Equation 1.


(1) $$\left[X^{t-T+1},X^{t-T+2},\cdots X^{t};G\right]\overset{\;f\left(.\right)\;}{\to }Y$$

### 3.2 Framework of MLFGCN

**Figure 3:**
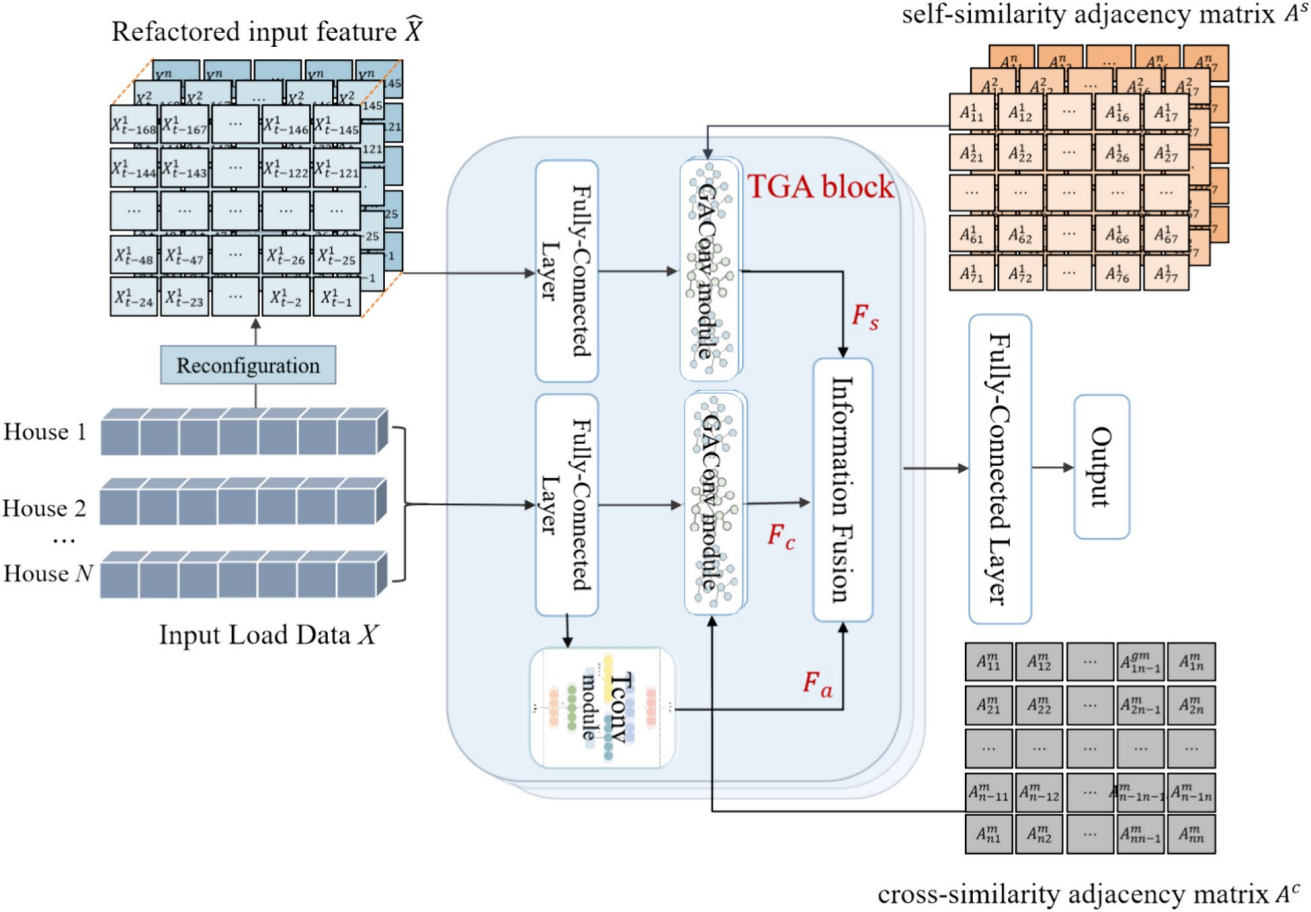
The framework of MLFGCN model.

**Figure 4:**
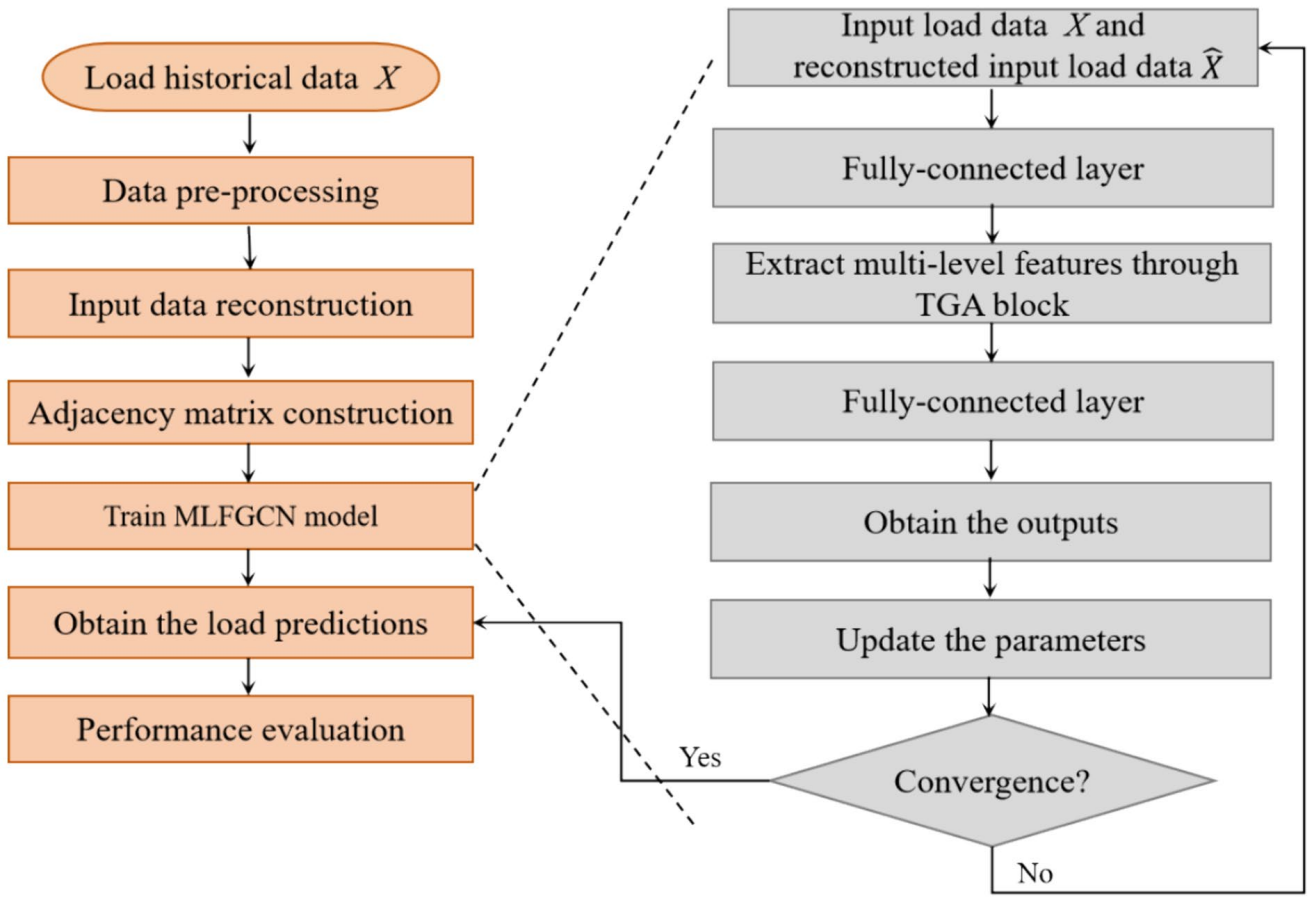
The whole process of MLFGCN.

Figure 3 shows the main framework of the proposed MLFGCN model. The network is composed of multiple stackable TConv-GAConv (TGA) blocks to capture the multi-level characteristics latent in load data. Each TGA block is composed of two parts: the graph convolution (GAConv) module and the temporal convolution (TConv) module. The whole process of MLFGCN is shown in Figure 4. Firstly, we reconstruct the input load series to better capture the periodic characteristics and the dependence of load series. Then, the reconstructed features of the input load series are input into the GAConv and TConv module to capture the internal dependence of a single load series and the interdependence between any two load series. Then, the high-dimensional features output from each module are fused at the information fusion layer, which is followed by a full connected layer to obtain the finally forecasting results.

### 3.3 Input load data reconstruction

For short-term residential load forecasting, the essence is to design a suitable model to predict the future load values of each residence by using the historical load data and related characteristics. Since the impact of inputs on model performance is crucial, selecting the appropriate inputs allows the model to better explore its intrinsic properties and obtain better output results. Therefore, further research on how to construct the input feature is necessary to improve the performance of the models.

The traditional input feature construction methods mainly including: (1) combining all the input data and related features in a stacked or tiled manner as the inputs of the model; (2) inputting the features of all residential loads separately to the forecasting model. The first type of the input construction hides the risk of “dimension explosion,” which can be effectively avoided though the second method. But the second construction method ignores the interaction characteristics of different load series. Based on the above considerations, we design an input feature reconstruction mechanism for load series. High-quality feature representation is obtained by constructing a multidimensional feature matrix, which effectively improves the model’s feature capture capability.

It is known that there are significant temporal correlations and periodic characteristics including daily-periodic characteristics and weekly-periodic characteristics in load series. Therefore, the one-dimensional input vector of the original load series of each residence is converted into a two-dimensional feature matrix with correlation. Due to the weekly periodicity characteristics, the historical loads of 7 days prior to the forecasting moment were chosen as inputs to the model in this study. Specifically, assuming that the load values at time-step t is to be predicted, the original input matrix of the $i_{th}$ house is $X_{t}^{i}$ , which can be written as Equation 2:


(2) $${\tilde{X}}_{t}^{i}=\left[X_{t-168}^{i},X_{t-167}^{i},\ldots ,X_{t-2}^{i},X_{t-1}^{i}\right]$$

The load values of a consecutive day are placed in one layer in order, and 1 week’s data are stacked in order. At this point, the converted input matrix of the $i_{th}$ house ${\hat{X}}_{t}^{i}$ can be written as Equation 3:


(3) $${\hat{X}}_{t}^{i}=f_{resh\mathrm{ape}}\left(X_{t}^{i}\right)=\left[\begin{array}{ccc} X_{t-168}^{i} & \cdots & X_{t-145}^{i}\\ \vdots & \ddots & \vdots \\ X_{t-24}^{i} & \cdots & X_{t-1}^{i} \end{array}\right]$$

where $f_{resh\mathrm{ape}}\left(\text{.}\right)$ means the reshape function.

**Figure 5:**
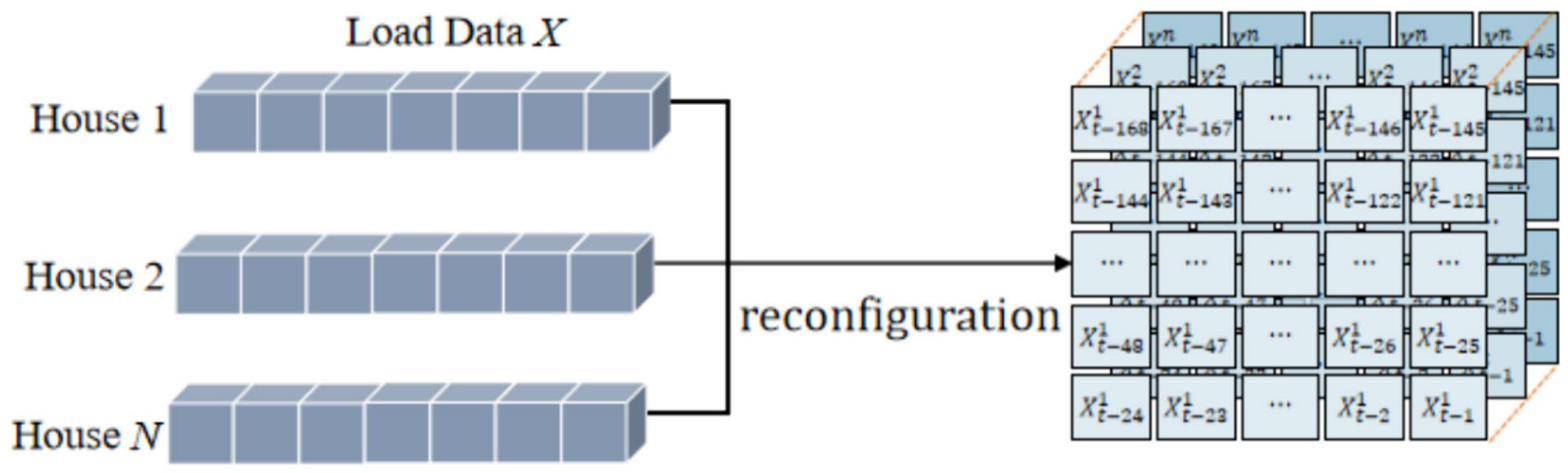
Input data reconstruction of MLFGCN.

There is also a strong correlation between the load patterns of multiple residential customers due to the similar living habits of users and external conditions. In this paper, according to the principle of alignment at the same time, the two-dimensional input matrices of different users are fused into three-dimensional feature matrices as the input of the model, as shown in Figure 5. The matrix distributes the data in a reasonable and orderly way, which keeps the input dimension within a reasonable range and helps the model better obtain the correlation between different houses. We obtain high quality input matrix by input feature construction, which effectively improves the performance of the model.

### 3.4 Graph attention convolution module

**Figure 6:**
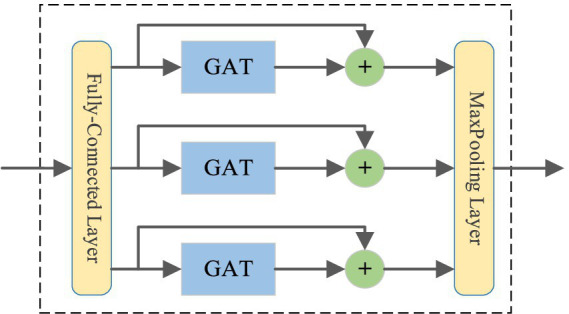
The structure of GAConv module.

The GAConv module aims to fuse the target node’s information with its neighbors’ features to obtain high-dimensional feature representation (Li et al., 2023). We design two GAConv modules to capture the internal dependence of a single load series and the interdependence between multiple load series, whose adjacency matrices are self-similarity adjacency matrix $A^{s}$ and cross-similarity adjacency matrix $A^{c}$ , respectively. Although the excellent ability of GCN in processing graph data has made breakthroughs in various fields (Yang et al., 2023), GCN cannot allocate different weights based on node importance, which is very important in the feature learning from electric load data. In GAConv module, we adopt graph attention network (GAT) to capture feature from different houses with different similarities. Figure 6 gives the structure of GAConv module. We input the feature matrix into the GAConv module and use the fully connected layer to reshape the inputs. GAT is introduced to calculate the hidden information corresponding to each node and dynamically capture the multi-level correlation features of different residences (Veličković et al., 2018). Graph convolution operation can be defined as Equation 4:


(4) $$Z=\tilde{A}XW$$

where $\tilde{A}\in {\mathbb{R}}^{N\times N}$ denotes the normalized adjacency matrix with self-loop, $X\in {\mathbb{R}}^{N\times d}$ denotes the input data, $W\in {\mathbb{R}}^{d}$ is the parameter matrix, $Z\in {\mathbb{R}}^{N}$ denotes the output signal.

#### 3.4.1 Adjacency matrix construction

**Figure 7:**
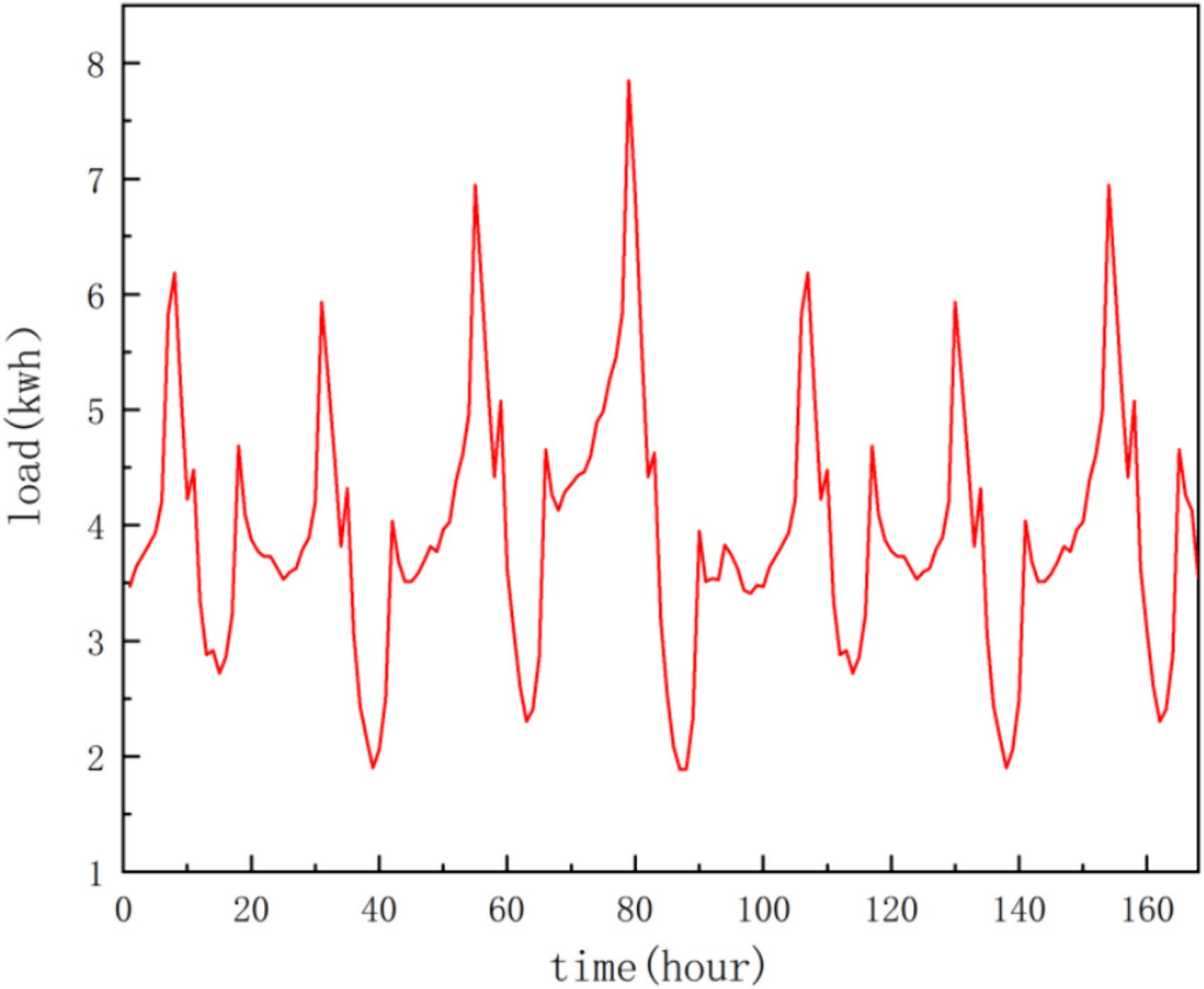
Weekly electricity load variation curve of a single house.

**Figure 8:**
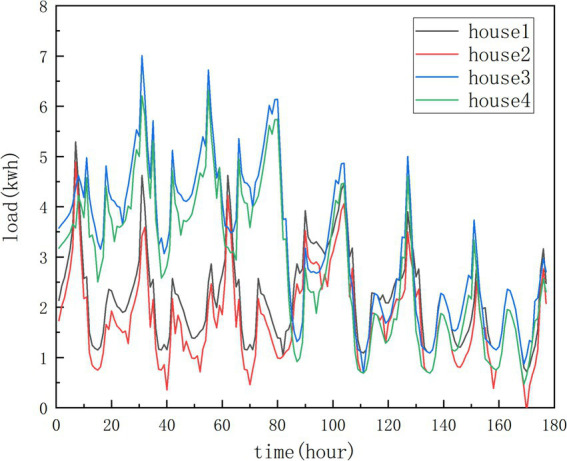
Weekly electricity load variation curve of four houses.

**Figure 9:**
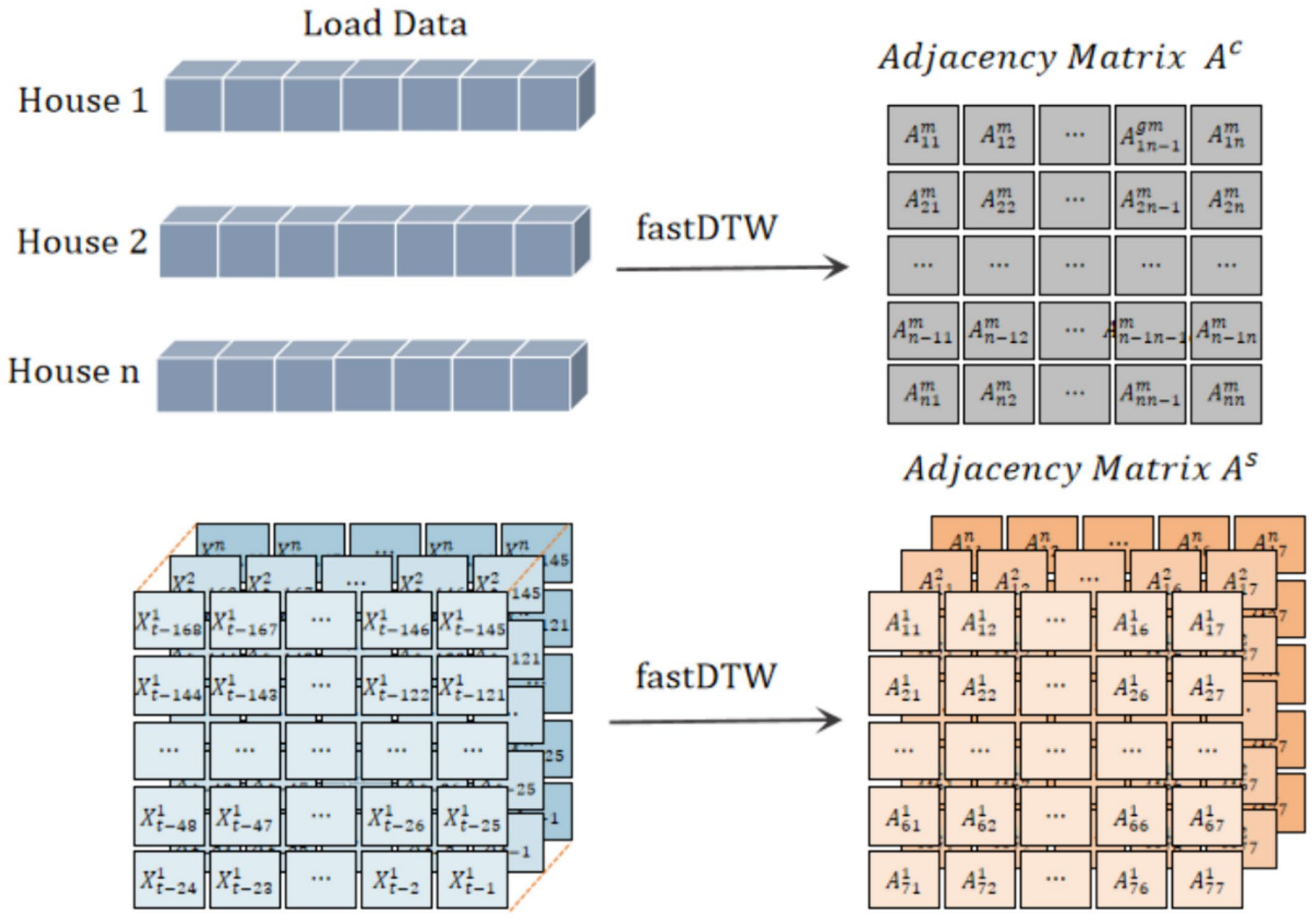
Generation of self-similarity adjacency matrix $A^{s}$ and cross-similarity adjacency matrix $A^{c}$ .

For graph neural networks, adjacency matrix is crucial. Multi-level correlation analysis on the features of different customers’ load data can effectively improve the forecasting performance. Load distribution of some houses maybe highly similar because the users’ similar living habits and external conditions. Figure 7 shows the electricity load curve of four residential houses in a week. It can be seen that there is strong periodicity for a single load curve. In addition, there are similar fluctuations over the same time period among the different load curves, as shown in Figure 8. The load curve fluctuations of house 1 and house 2, house 3 and house 4 are very similar, with peak and valley loads appearing approximately simultaneously. Therefore, merely utilizing the correlation between geographical locations cannot accurately obtain the dependence between various load data. In this paper, we use two types of adjacency matrices to learn multi-level dependence (as shown in Figure 9), that is, the self-similarity adjacency matrix $A^{s}$ and the cross-similarity adjacency matrix $A^{c}$ . $A^{s}$ is obtained by calculating the internal similarity of a single load series and $A^{c}$ is obtained by calculating the similarity between multiple load series.

Many existing methods can be used to calculate the similarity of time series. In our proposed model, fastDTW algorithm is used to extract the similarity adjacency matrices of individual houses and multiple houses, respectively. FastDTW algorithm is an efficient way to calculate the similarity between two time series by automatically warping them, especially suitable for time series of different lengths and rhythms. The specific calculation process is shown in Algorithm 1. Compared with the traditional Euclidean distance matrix, fastDTW distance matrix can more accurately describe the consistency of each user.

##### ALGORITHM 1 The calculation process of fastDTW

Input: *A* $=\left(a_{1},\cdots a_{m}\right)\in {\mathbb{R}}^{m\times d}$ , $B=\left(b_{1},\cdots b_{n}\right)\in {\mathbb{R}}^{n\times d}$ , searching length *L*

1. for i=1,2…m do
2. for $j=\max\left(0,i-L\right),\ldots ,\min\left(m,i+L+1\right)$ do
3.
$D_{i,j}=|A_{i}-B_{j}|$

4. if i=0,j=0 then $M_{s}\left(i,j\right)=D_{i,j}$
5. else if *i* =0 then $M_{s}\left(i,j\right)=D_{i,j}+M_{i,j-1}$
6. else if *j* =0 then $M_{s}\left(i,j\right)=D_{i,j}+M_{i-1,j}$
7. else if j=i−L then

$M_{s}\left(i,j\right)=D_{i,j}+\min\left(M_{i-1,j-1},M_{i-1,j}\right)$

8. else if j=i+L then

$M_{s}\left(i,j\right)=D_{i,j}+\min\left(M_{i-1,j-1},M_{i,j-1}\right)$

9. else $M_{s}\left(i,j\right)$ = $D_{i,j}+\min\left(M_{i-1,j-1},M_{i,j-1},M_{i-1,j}\right)$
10. end
11. end
12. return $\mathrm{dis}\left(A,B\right)=M_{s}$

#### 3.4.2 Graph attention network

GAT can assign different weights to the input features and highlight the more critical features for more effectively information aggregating. This correlation in load data is captured synchronously by several parallel GAT blocks to increase the prediction accuracy of the model. It can directly reflect the connections between different residences thanks to the construction of multidimensional feature matrix. Thus, two layers of convolution is sufficient to aggregate the valuable information of the neighboring nodes. Given the node feature $h=\left\{h_{1},h_{2},\ldots ,h_{N}\right\}$ , the attention coefficients between two neighbor nodes $v_{i}$ and $v_{j}$ can be expressed as Equation 5:


(5) $$e_{ij}=\sigma \left(Wh_{i},Wh_{j}\right),j\in N_{i}$$

where W is weight matrix, $j\in N_{i},N_{i}$ is a set of neighbor nodes of node $v_{i}$ . In order to make attention coefficient easier to calculate and compare, we introduced softmax function to normalize them. It can be written as Equation 6.


(6) $$a_{ij}=\mathrm{softmax}\left(e_{ij}\right)=\frac{\exp\left(e_{ij}\right)}{\Sigma_{k\in N_{i}}\exp\left(e_{ik}\right)}$$

Then, the features are weighted and summed up using attention coefficients.


(7) $$h_{i}=\sigma \left(\underset{j\in N_{i}}{\sum }\alpha_{ij}W^{k}h_{j}\right)$$

In order to stabilize the learning process of self attention, we use multi-head attention to obtain rich representations. Specifically, *K* independent attention mechanisms execute Equation 7 and then concatenate their features together to achieve the final results.


(8) $${\hat{h}}_{i}={∥}_{k=1}^{K}\sigma \left(\underset{j\in N_{i}}{\sum }\alpha_{ij}W^{k}h_{j}\right)$$

In Equation 8, || represents concatenation. The output of GAT can be written as Equation 9:


(9) $$Z^{l}=\tilde{A}Z^{l-1}W^{l}=W^{l}\hat{h}$$

where $Z^{0}=X$ , $\tilde{A}=A^{s}$ for self-similarity feature leaning and $\tilde{A}=A^{c}$ for cross-similarity feature leaning. Here, we use MaxPooling to manipulate the connections of each hidden state. The output $F_{s}$ of self-similarity feature learning module and the output $F_{c}$ of the cross-similarity feature learning module can be written as Equations 10 and 11, respectively:


(10) $$F_{s}=\mathrm{softmax}\left(A^{s}\mathrm{Relu}\left(A^{s}\hat{X}W_{s}^{1}\right)W_{s}^{2}\right)$$

(11) $$F_{c}=\mathrm{softmax}\left(A^{c}\mathrm{Relu}\left(A^{c}XW_{c}^{1}\right)W_{c}^{2}\right)$$

### 3.5 Temporal convolution module

**Figure 10:**
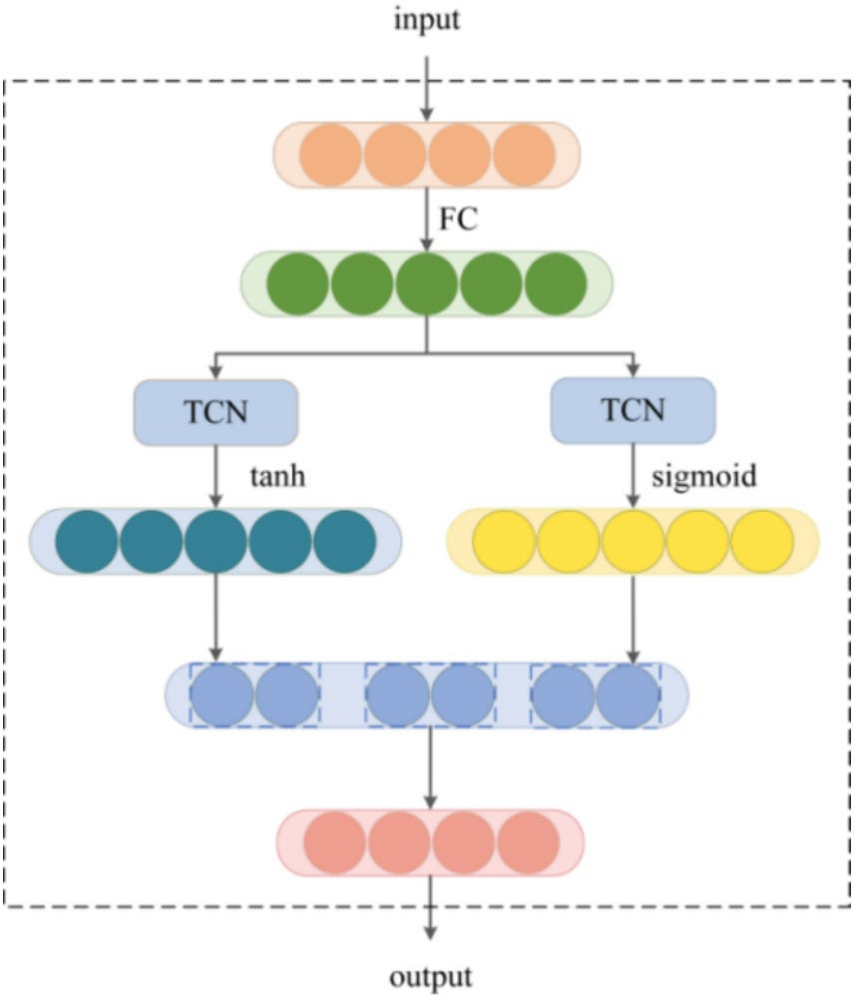
The structure of the TConv module.

The TConv module is designed based on gated TCN to obtain long-term temporal dependencies of the load series. As shown in Figure 10, we design a gating mechanism to filter out weak connections and obtain optimized features. Compared to RNN-based neural networks, TCN reduces parameter complexity by using the expanded causal convolution operation. The window size of TCN grows exponentially with the number of layers, which allows a larger receptive field with only a few convolution operations. Let *X* be the input, the output $F_{a}$ of the gated TCN can be expressed as Equation 12:


(12) $$F_{a}=\tan h\left({TCN}_{a}\left(Χ\right)\right)⊙\sigma \left({TCN}_{b}\left(Χ\right)\right)$$

where tanh and σ are two different activation functions, ${TCN}_{a}\left(\text{.}\right)$ and ${TCN}_{b}\left(\text{.}\right)$ are two TCNs, ⊙ represents element-wise product.

### 3.6 Information fusion

After the above calculation process, high-dimensional features from GAConv module and TConv module are obtained. Then, we effectively fuse these valuable features to improve the accuracy of load forecasting. We adopt addition for information aggregation to generate the final predictions. The specific calculation process can be written as Equation 13:


(13) $$Y=\alpha F_{a}+\beta F_{c}+\gamma F_{s}$$

where α , β and γ are the learnable parameters.

Finally, we summarize the proposed MLFGCN as shown in Algorithm 2.

#### ALGORITHM 2 MLFGCN for short-term load forecasting

Input: The load observed data $X=\left[X^{1},X^{2},\cdots X^{N}\right]\in {\mathbb{R}}^{N\times T}$

1. Generate reconstructed input load data $\hat{X}$ from *X*;
2. Generate self-similarity adjacency matrix $A^{s}$ and cross-similarity adjacency matrix $A^{c}$ for the load graph G through Algorithm 1;
3. Get the periodic feature $F_{s}$ by GAConv module using self-similarity adjacency matrix $A^{s}$ ,
$F_{s}=\mathrm{softmax}\left(A^{s}\mathrm{Relu}\left(A^{s}\hat{X}W_{s}^{1}\right)W_{s}^{2}\right)$ ;
4. Get the interdependent feature $F_{c}$ by GAConv module using cross-similarity adjacency matrix $A^{c}$ , $F_{c}=\mathrm{softmax}\left(A^{c}\mathrm{Relu}\left(A^{c}XW_{c}^{1}\right)W_{c}^{2}\right)$ ;
5. Get the temporal feature $F_{a}$ by TConv module, $F_{a}=\tanh\left(TCN\mathrm{a(X)}\right)⊙\sigma \left(TCN\mathrm{b(X)}\right)$
6. Get the output Y by integrating $F_{a}$ , $F_{c}$ and $F_{s}$ , $Y=\alpha F_{a}+\beta F_{c}+\gamma F_{s}$ ;
7. Return the output;
8. Calculate the loss of MLFGCN

### 3.7 Loss function of MLFGCN

There are noise and outliers in the electric load data, which have a negative impact on the prediction results. To address this issue, we select Huber Loss as the loss function. Huber loss function is widely used in regression problems that combines the advantages of mean square error and mean absolute error. Huber loss function is more robust when dealing with outliers and can effectively reduce the influence of outliers on the model. It can be written as Equation 14:


(14) $$L\left(\hat{Y},Y\right)=\{\begin{array}{c} \frac{1}{2}{\left(\hat{Y}-Y\right)}^{2},|\left(\hat{Y}-Y\right)|\leq \delta \\ \delta |\hat{Y}-Y|-\frac{1}{2}\delta^{2},|\hat{Y}-Y|>\delta \end{array}$$

where δ is hyperparameter to control sensitivity of the loss. Y and $\hat{Y}$ are the real load values and the predictions, respectively.

## 4 Experiment and result analysis

### 4.1 Datasets

In this section, we validate the superiority of the proposed MLFGCN model on several real-world cases and analyze the experimental results.

**Case 1:** This experimental dataset is from OpenEI (National Renewable Energy Laboratory, 2014), which includes loads for all major types of residential and commercial buildings across all climate regions in the United States. The dataset is collected at 1-h resolution. We demonstrate the effectiveness of the algorithm by randomly selecting 15 houses in Los Angeles (LA).

**Case 2:** This experimental dataset is from a real power grid in the United States provided by Iowa State University (Bu et al., 2019). The power grid contains 240 nodes from three feeders including 17 nodes in Feeder_A dataset, 60 nodes in Feeder_B, and 163 nodes in Feeder_C. The data of each node are the measurements from the users’ smart meters, which is collected at 1-h resolution.

Table 2 summarizes the characteristics of these datasets. We first preprocess the sample data and use *z*-score normalization to normalize the load data.


(15) $${Χ}^{z}=\frac{Χ-\mathrm{mean}\left(Χ\right)}{std\left(Χ\right)}$$

In Equation 15, $\mathrm{mean}\left(Χ\right)$ and $std\left(Χ\right)$ are the mean value and the standard deviation of the historical load series, respectively.

**Table 2:** Summary of the experimental datasets.

	Dataset	Sample size	Number of houses
Case 1	LA	8,760	15
Case 2	Feeder_A	8,760	17
	Feeder_B	8,760	60
	Feeder_C	8,760	163
	Feeder_Sum	8,760	240

### 4.2 Evaluation metrics

The mean absolute error (MAE), mean absolute percentage error (MAPE) and root mean square error (RMSE) are used to evaluate the accuracy of the proposed model. For them, the lower the value, the better the forecasting performance. MAE, MAPE and RMSE are defined as:


(16) $$MAE=\frac{1}{n}\overset{n}{\underset{t=1}{\sum }}|y_{t}-{\hat{y}}_{t}|$$

(17) $$\mathrm{MAPE}=\frac{1}{n}\overset{n}{\underset{t=1}{\sum }}|\frac{y_{t}-{\hat{y}}_{t}}{y_{t}}|\times 100\%$$

(18) $$\mathrm{RMSE}=\sqrt{\overset{n}{\underset{t=1}{\sum }}{\left(y_{t}-{\hat{y}}_{t}\right)}^{2}}$$

In Equations 16, 17 and 18, $y_{t}$ and ${\hat{y}}_{t}$ refer to the real load values and the predicted load values of the model at time step *t*, respectively. n is the number of samples.

### 4.3 Baselines and experimental settings

In this paper, five load forecasting models are selected as the baselines to validate the performance of the proposed MLFGCN model. The baseline models include mainstream load forecasting methods, among which SVR belongs to statistical methods, LSTM is the most commonly used time series forecasting method, CNN-GRU is spatiotemporal load forecasting method based on Euclidean distance, STGCN Multi-hop and Ada GWN are spatiotemporal load forecasting methods based on non-Euclidean distance.

- SVR: Support vector regression (SVR) is a regression method based on support vector machine (SVM), commonly used for time series prediction.
- LSTM: Long short-term memory network (LSTM), which performs well in long time series forecasting.
- CNN-GRU: CNN-GRU model, which is a hybrid model combing CNN and GRU for short-term residential load forecasting.
- STGCN Multi-hop: Spatial–temporal graph convolutional networks (STGCN) with the input graph nodes more than one hop away as neighbors, which is a spatiotemporal model to predict the load consumption values for each customer (Cheung et al., 2021).
- Ada-GWN: Spatial–temporal residential short-term load forecasting network based on Graph WaveNet framework (Lin et al., 2021).

We divide the experimental dataset into training set, validation set and test set in a ratio of 6:2:2. To make a fair comparison with the baseline models, all forecasting models used for experiments are conducted with Pytorch framework on servers under the same configuration. We set the search length of the fastDTW to be 24. Huber loss is selected as the loss function and the Adam optimizer is used for optimization. The learning rate is set to 0.001, the epoch is 200, and the batch size is 32. The parameter settings are the same for all models. We set three TGA blocks for load forecasting, which contains an independent TConv block and two GAConv blocks. Each experimental dataset was evaluated more than 10 times to ensure the accuracy of the results.

### 4.4 Experimental results and analysis

The experiments are divided into three parts, and the experimental results are discussed in three aspects: performance analysis of the proposed MLFGCN model, impact analysis of the number of houses and ablation experiments. The experimental results show that the proposed MLFGCN model has better prediction performance compared with baseline models.

#### 4.4.1 Performance analysis of MLFGCN

We first evaluate the performance of MLFGCN on case 1. The experimental results are shown in Table 3.

**Table 3:** Performance comparison of load forecasting models on the LA dataset.

	LA dataset		
	MAE (KWh)	MAPE (%)	RMSE (KWh)
SVR	0.86	10.49	1.83
LSTM	0.76	9.74	1.43
CNN-GRU	0.64	9.31	0.97
STGCN Multi-hop	0.39	8.73	0.62
Ada-GWN	0.28	8.16	0.57
MLFGCN	**0.25**	**7.58**	**0.50**

Results presented in bold are the best performance.

**Figure 11:**
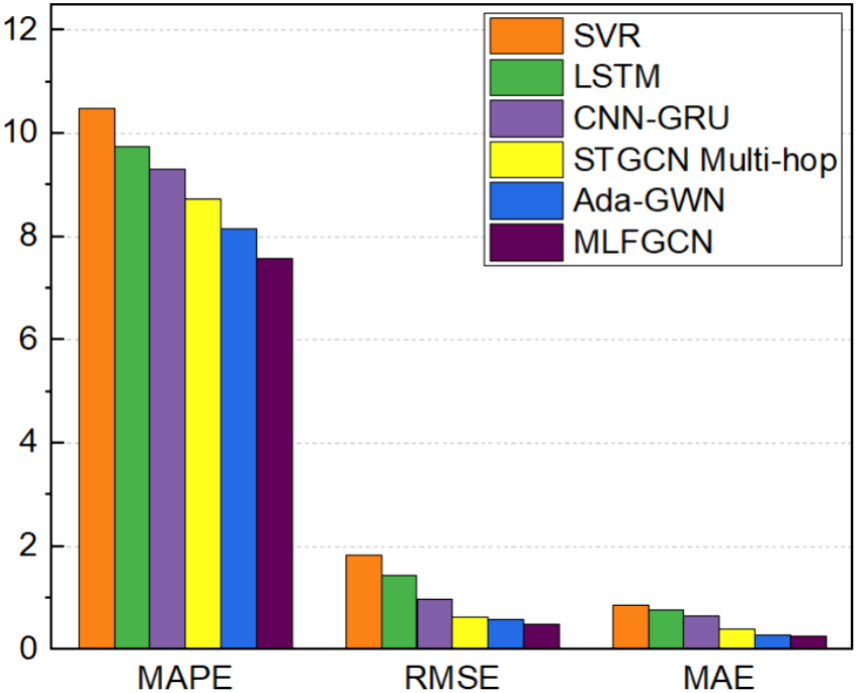
Performance comparison of load forecasting on the LA dataset.

Figure 11 visualizes the results for three metrics MAPE, RMSE, and MAE, respectively. It can be seen that, compared with the traditional SVR model, MAE, MAPE and RMSE values of MLFGCN model decreases by 70.93, 27.74, and 72.68%. Although SVR is widely used in time series prediction tasks, there are still limitations when dealing complex nonlinear relationships. At the same time, MLFGCN has higher forecasting accuracy compared with the models dedicated to temporal prediction such as LSTM, because only learning temporal features cannot capture valuable information comprehensively. CNN-GRU, STGCN Multi-hop models and Ada-GWN all consider the spatial–temporal features in load data, but there is still a big gap between them. STGCN Multi-hop and Ada-GWN achieved better prediction results than CNN-GRU because spatial modeling based on non-Euclidean distance is more suitable for power load data. Even so, compared with Ada-GWN, MAE, MAPE and RMSE of MLFGCN model decreases by 12, 7.65, and 14%, respectively. In summary, MLFGCN model proposed in this paper can effectively utilize historical load data information to accurately predict future load values and is superior to the baseline models.

#### 4.4.2 Impact analysis of the number of houses

To analyze the impact of the number of houses on model performance, a real-word dataset from Iowa, USA, was selected for this study. The dataset contains load data of 240 units from three feeders with 17, 60, and 163 houses, respectively. Three baseline models, CNN-GRU, STGCN Multi-hop, and Ada-GWN are selected as the comparison models. The results are shown in Table 4.

**Table 4:** Comparative experimental results on the dataset of case 2.

Datasets	Metrics	CNN-GRU	STGCN Multi-hop	Ada-GWN	MLFGCN
Feeder_A	MAE (KWh)	1.71	1.72	1.66	**1.65**
	MAPE (%)	29.27	28.13	27.34	**26.98**
	RMSE (KWh)	4.36	3.92	**3.72**	3.80
Feeder_B	MAE (KWh)	1.83	1.71	1.70	**1.61**
	MAPE (%)	29.56	27.32	26.98	**26.18**
	RMSE (KWh)	4.72	3.94	4.05	**3.51**
Feeder_C	MAE (KWh)	1.98	1.68	1.57	**1.45**
	MAPE (%)	29.32	27.29	26.31	**25.23**
	RMSE (KWh)	3.79	3.65	3.35	**3.20**
Feeder_Sum	MAE (KWh)	1.81	1.62	1.48	**1.33**
	MAPE (%)	28.54	27.05	26.24	**25.63**
	RMSE (KWh)	3.66	3.48	3.29	**3.16**

Results presented in bold are the best performance.

**Figure 12:**
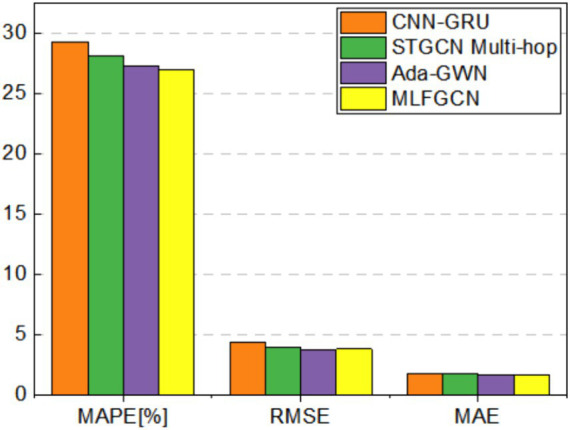
Comparative experimental results on Feeder_A.

**Figure 13:**
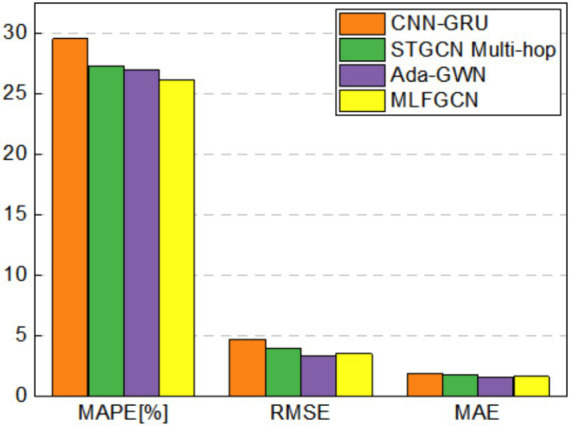
Comparative experimental results on Feeder_B.

**Figure 14:**
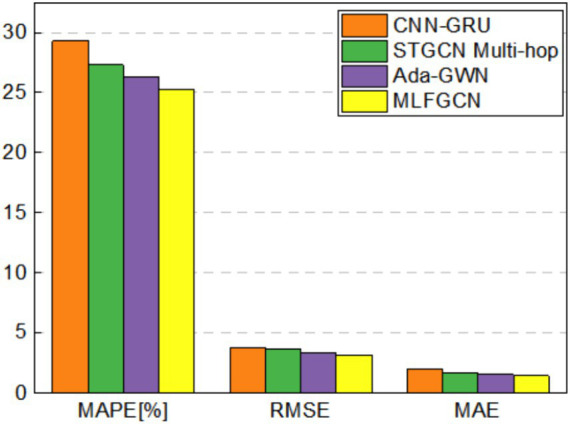
Comparative experimental results on Feeder_C.

**Figure 15:**
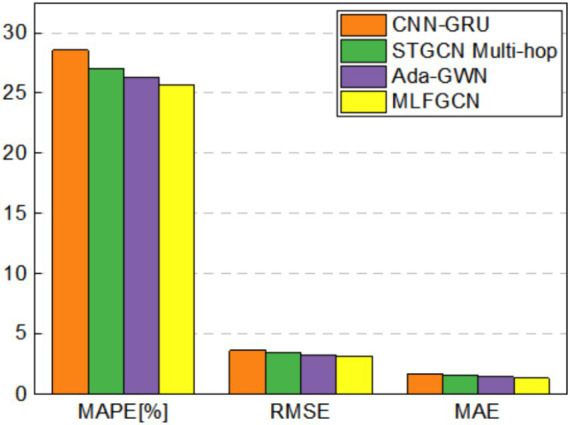
Comparative experimental results on Feeder_Sum.

Figures 12–15 visualize the experimental results on the datasets of the three feeders: Feeder_A, Feeder_B, and Feeder_C. Feeder_Sum is all load data for the three subregions. It can be seen that the CNN-GRU model performs well in the Feeder_A, with a MAE value only 5.8% higher than MLFGCN. However, in Feeder_B, Feeder_C, and Feeder_Sum, where the number of houses is relatively high, the gap between MLFGCN and the other baselines will become larger and larger as the number of houses increases. Similar to MLFGCN, the prediction accuracy of Ada-GWN also continuously improves with the increase of the number of houses. It can be seen that CNN-GRU is more suitable for the case with a few houses. When the number of houses is small, CNN-GRU has about the same predictive accuracy as MLFGCN. The values of MAE, MAPE, and RMSE of STGCN Multi-hop are stable around 1.7, 27.5, and 3.6 for different number of houses, which indicates that STGCN Multi-hop is minimally affected by the number of houses.

For the MLFGCN model proposed in this paper, the predictive performance advantage is not significant when the number of houses is small. As the number of houses continues to increase, the performance advantages of MLFGCN gradually become apparent. Especially on the Feeder_Sum dataset, where MAE, MAPE, and RMSE values of MLFGCN model decreases by 26.52, 10.20, and 13.66% compared to CNN-GRU, and decreases by 17.90, 5.25, and 9.20% compared to STGCN Multi-hop. As the number of houses increases, the MLFGCN model can learn richer features by comparing and analyzing load series with similar patterns, which can improve the generalization ability and prediction accuracy of the forecasting model.

#### 4.4.3 Ablation experiments

This section analyzes the necessity of input feature construction and the effectiveness of each part of the proposed model, respectively. The experimental results show that each part of MLFGCN is effective on the prediction results.

Comparison experiments were first conducted on the Feeder_Sum dataset to validate the input feature construction, and experimental results are shown in Table 5. The results show that the MAE, MAPE, and RMSE values of the model with input feature reconstruction decreased by 13.04, 8.82, and 21.65%, respectively, which indicates that modeling the raw input data can improve the forecasting accuracy.

**Table 5:** Impact of input feature construction on predictive performance of MLFGCN.

	Feeder_Sum dataset		
	MAE (KWh)	MAPE (%)	RMSE (KWh)
MLFGCN	**1.38**	**24.91**	**3.22**
MLFGCN without input feature reconstruction	1.56	27.32	4.11

Results presented in bold are the best performance.

Then, we verify the effect of adjacency matrix construction, TConv module and GAConv module on the forecasting performance. We design three variants named MLFGCNI, MLFGCNII, and MLFGCNIII, whose specific configuration are shown in Table 6. MLFGCNI is designed to replace the adjacency matrix construction of MLFGCN with an adaptive adjacency matrix. MLFGCNII and MLFGCNIII are variants of MLFGCN with TConv module or GAConv module removed, respectively, while the rest remain unchanged. The ablation experiments were conducted on both LA and Feeder_Sum datasets. The results are shown in Table 7.

**Table 6:** Configuration of models for ablation experiments.

	Components of MLFGCN		
	Adjacency matrix construction	TConv	GAConv
MLFGCNI		✔	✔
MLFGCNII	✔		✔
MLFGCNIII	✔	✔	
MLFGCN	✔	✔	✔

**Table 7:** Ablation experiments on dataset LA and Feeder_Sum.

	LA dataset			Feeder_Sum dataset		
	MAE (KWh)	MAPE (%)	RMSE (KWh)	MAE (KWh)	MAPE (%)	RMSE (KWh)
MLFGCNI	0.31	8.24	0.51	1.51	25.17	3.46
MLFGCNII	0.35	8.47	0.45	1.48	24.91	3.39
MLFGCNIII	0.48	8.37	0.56	1.69	25.31	3.53
MLFGCN	**0.27**	**7.97**	**0.45**	**1.48**	**24.91**	**3.22**

Results presented in bold are the best performance.

From the results, we can see that the performance of MLFGCNII is better than MLFGCNIII, which indicates that the GAConv module is more effective than the TConv module. The graph attention network in the GAConv module can better capture the local and global correlation features in the load data. The forecasting results of MLFGCNI is better than MLFGCNII but inferior to MLFGCN, which demonstrates both GAConv module and TConv module can improve the performance of the MLFGCN model. Meanwhile, the experimental results indicate that the adjacency matrix learned through the fastDTW algorithm can effectively capture the potential interdependence relationships in the load data to obtain more accurate prediction results.

#### 4.4.4 Training efficiency

We compare the computation cost of the spatiotemporal forecasting models: CNN-GRU, STGCN Multi-hop, Ada-GWN and the proposed MLFGCN on LA dataset. The results are shown in Table 8. During the training phase, MLFGCN outperforms CNN-GRU and Ada-GWN. Thanks to the temporal convolution structure, STGCN Multi-hop is slightly better than MLFGCN, but its prediction performance is slightly worse than MLFGCN. During the inference phase, MLFGCN is comparable with Ada-GWN, and slightly faster than CNN-GRU. It is worth noting that there is not significant difference in the inference time of each model when making one-step predictions. From the perspective of both predictive performance and computation cost, MLFGCN is still a very excellent forecasting model.

**Table 8:** The computation time on the LA dataset.

Models	Computation Time	
	Training time (s/epoch)	Inference time (s)
CNN-GRU	128.44	9.53
STGCN Multi-hop	58.30	7.78
Ada-GWN	70.78	8.64
MLFGCN	62.34	8.56

**Figure 16:**
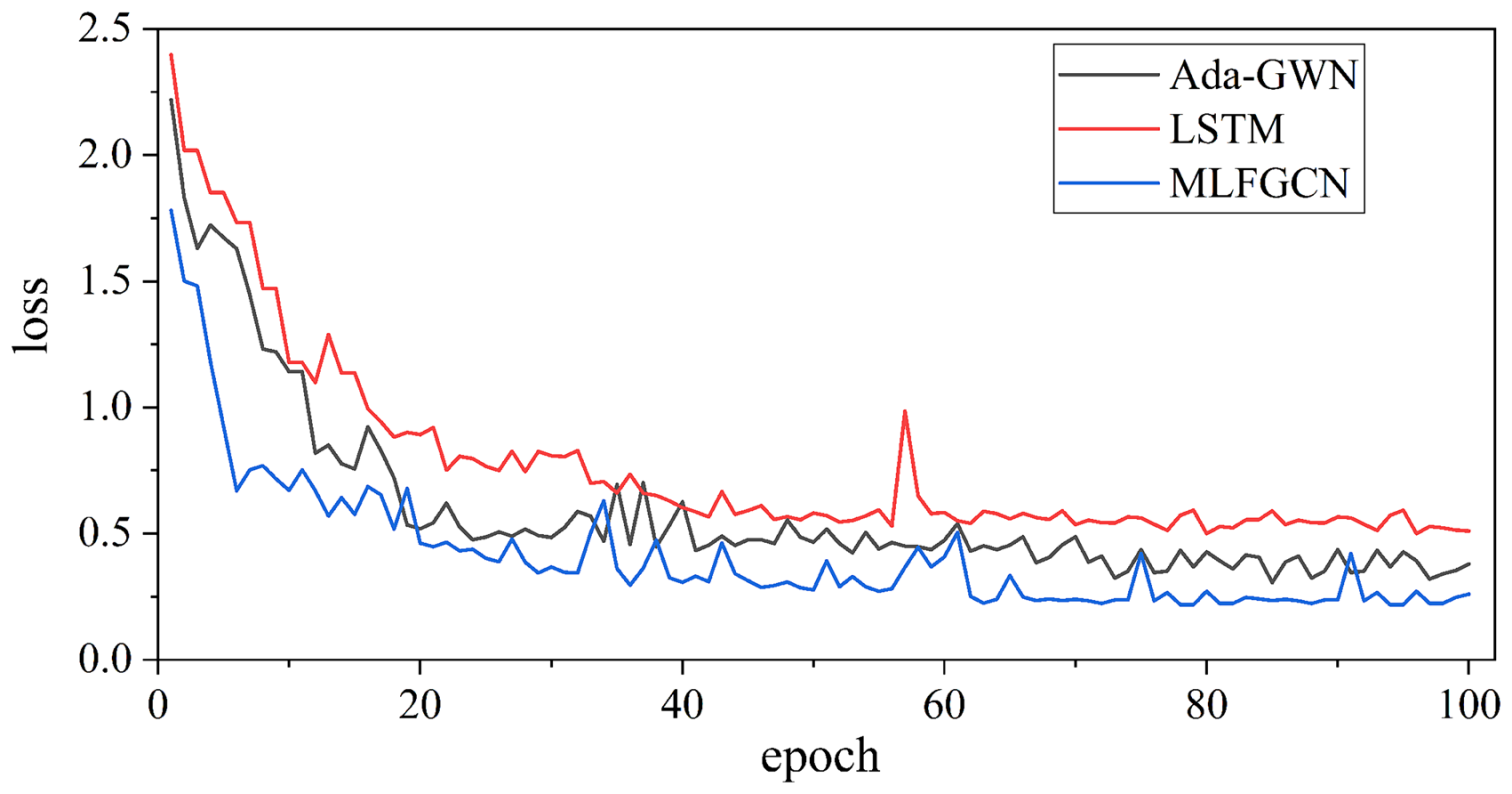
The convergence curve of different models on LA dataset.

In order to further investigate the performance of MLFGCN, we compare the training loss convergence process of the models. We selected LSTM and AGWN as the baselines, where LSTM is a load forecasting method based on time series prediction, and AGWN is based on spatiotemporal prediction. As shown in Figure 16, the training loss of all models rapidly decreases with the increase of epochs and eventually reaches convergence. Compared to the baseline models, our proposed model can achieve easier convergence. Thanks to the special design of TGA blocks, our model allows parallel learning of temporal and spatial features to improve time efficiency.

## 5 Conclusion

Residential load forecasting is a challenging task due to the random fluctuations caused by complex correlations and individual differences. This paper proposes a novel multi-level feature fusion model based on graph attention temporal convolutional network (MLFGCN) for short-term residential load forecasting. The proposed MLFGCN model fully consider the potential long-term dependencies of a single load series and the correlations between multiple load series. TCN network with gating mechanism is introduced to learn potential long-term dependencies in the original load series. In addition, we design two graph attentive convolutional modules to capture potential multi-level dependencies in load data. Finally, the output of each module are fused through an information fusion layer to obtain the highly accurate forecasting results. We conduct validation experiments on two real-world datasets to demonstrate the superiority of MLFGCN.

Although MLFGCN performs well in short-term residential load forecasting, its accuracy will continue to decline as the prediction scale increases. At the same time, the training complexity of MLFGCN is still relatively high. In the next step, we will focus on how to improve the long-term predictive ability of the model and how to reduce training complexity. In addition, load probability prediction is also crucial for power scheduling, and how to complete probability prediction based on point prediction is also our key work.

## Data Availability

The datasets presented in this study can be found in online repositories. The names of the repository/repositories and accession number(s) can be found below: https://www.osti.gov/biblio/1788456.
